# The oncogenic role of mir-21 in cervical cancer: linking epithelial–mesenchymal transition to stromal remodeling

**DOI:** 10.3389/fonc.2026.1814515

**Published:** 2026-08-03

**Authors:** Ingrid Galvis, Aiman Hafeez, Jare Philip, Mary Salvatore

**Affiliations:** 1 Department of Radiology, Jacobi Medical Center, New York City, NY, United States; 2 Department of Radiology, Albert Einstein College of Medicine, New York City, NY, United States

**Keywords:** cancer invasion and metastasis, cervical cancer, epithelial to mesenchymal transformation (EMT), human papillomavirus (HPV), miR-21, stromal remodeling

## Abstract

**Background:**

Cervical cancer remains a leading cause of cancer mortality worldwide, driven primarily by persistent infection with oncogenic human papillomavirus (HPV). Epithelial–mesenchymal transition (EMT) is a critical process in tumor invasion and metastasis, and miR-21 has been identified as a frequently upregulated oncomiR whose locus at 17q23.2 overlaps a common HPV16 integration site and which simultaneously targets multiple tumor suppressors, positioning it as a candidate convergent oncomiR with proposed regulatory roles in both processes.

**Objective:**

This review synthesizes current knowledge on the oncogenic role of miR-21 in cervical cancer, with particular emphasis on its dual function in EMT and stromal remodeling.

**Methods:**

We conducted a narrative review of published studies identified through PubMed, Scopus, Web of Science and Embase. Included studies were heterogeneous in design, comprising *in vitro* cell-line experiments, tissue-based clinical studies, and mixed models; findings were interpreted according to their level of evidence.

**Key findings:**

miR-21 is frequently upregulated in HPV-positive cervical cancer cell lines and tissue specimens, where it has been shown — predominantly in preclinical models — to suppress tumor suppressors, downregulate epithelial markers, and upregulate mesenchymal markers. It is also enriched in tumor-associated fibroblasts in experimental models, where it has been associated with fibroblast activation and extracellular matrix remodeling. Preclinical inhibition of miR-21 reverses EMT markers and improves chemosensitivity in cell-line models.

**Conclusion:**

This review integrates evidence across miR-21, EMT, and stromal remodeling in cervical cancer, proposing a conceptual framework in which miR-21 functions as a convergent regulator linking tumor cell EMT to fibroblast-driven stromal remodeling— a synthesis not previously proposed in the literature. The current evidence, mainly based on *in vitro* and cell-line studies conducted in Asian populations, supports the biological plausibility of this role; however, it has not yet established a causal relationship in human cervical cancer. Patient-level validation in diverse, well-characterized cohorts is needed before miR-21 can be considered a clinically actionable biomarker or therapeutic target.

## 1 Introduction

Cervical cancer remains one of the leading causes of cancer-related morbidity and mortality worldwide. Each year, around 500,000 new cases are diagnosed, with the highest burden in low- and middle-income countries (1). In the United States, 11,500 cases of cervical cancer occur annually, resulting in 4,000 deaths (2–4). The primary causal factor is persistent infection with oncogenic human papillomavirus (HPV), a common sexually transmitted infection that in the majority of cases, clears on its own. Persistent HPV infection in the cervical basal epithelial layers results in progressive oncogenic cellular changes that serves as precursors to cervical cancer (1). Despite advances in HPV vaccination and screening, locally invasive and metastatic disease remains a leading cause of cervical cancer mortality, and its molecular mechanisms of invasion are incompletely understood (5, 6).

At the molecular level, chronic expression of HPV *E6* and *E7* oncogenes promotes tumor progression by inducing genetic and epigenetic instability, including microRNA dysregulation (6–9). Among these, miR-21 is upregulated in cervical cancer and other malignancies, where it regulates gene expression post-transcriptionally and targets tumor suppressors such as PDCD4 and PTEN (5, 10, 11). Its expression is particularly elevated in HPV16+ and HPV18+ cervical cancer cell lines, correlating with histological stage (5). In addition to promoting proliferation and inflammation-associated carcinogenesis via NF-κB and IL-6 signaling, miR-21 is aberrantly expressed in the tumor stroma, where fibroblast activation has been associated with tumor growth, angiogenesis, and myofibroblast differentiation in preclinical models (12, 13).

Prior reviews have examined the diagnostic and prognostic value of miR-21 in cervical cancer, but none has proposed a unified framework in which miR-21 may act as a convergent regulator linking epithelial–mesenchymal transition (EMT) to stromal remodeling (5, 7, 14). Given that local invasion and metastasis remain the principal determinants of poor prognosis, this mechanistic integration is clinically relevant. This review proposes and critically evaluates that framework, synthesizing the current literature across cellular, stromal, and translational studies while highlighting its strengths, limitations, and potential implications for biomarker development and therapeutic targeting.

## 2 Methods

**Figure 1:**
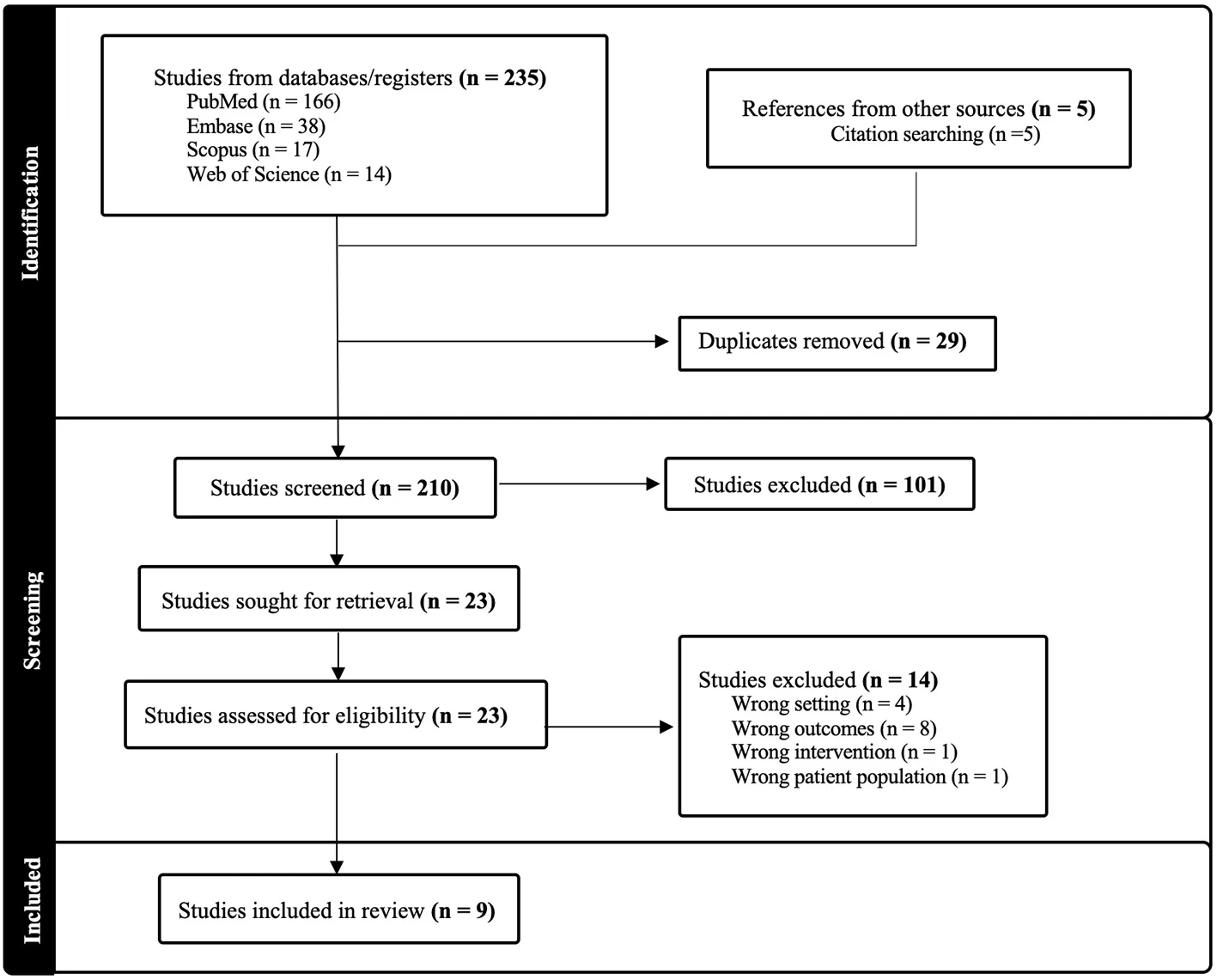
PRISMA flow diagram of study identification, screening, eligibility assessment, and inclusion. Studies were identified through searches of PubMed, Embase, Scopus, and Web of Science, supplemented by citation screening. After duplicate removal, records were screened according to predefined eligibility criteria. Full-text articles were assessed for relevance to the role of miR-21 in cervical cancer, epithelial–mesenchymal transition (EMT), and stromal remodeling. Nine studies met the inclusion criteria and were incorporated into the primary evidence synthesis.

This review was conducted as a narrative, hypothesis-generating synthesis; the PRISMA flow diagram is included to provide transparency regarding the database search and study selection process, consistent with the review’s narrative, hypothesis-generating aims. We searched four databases—PubMed, Scopus, Web of Science, and Embase—from database inception through 2025 using combinations of the terms “microRNA-21”, “miR-21”, “epithelial–mesenchymal transition”, “EMT”, “cervical cancer”, “cervical carcinoma”, and “uterine cervical neoplasms”, adapted to each database’s vocabulary and syntax. A total of 240 records were identified, including 235 from database searching and 5 through citation screening. After removal of 29 duplicates, 210 records were screened by title and abstract, of which 101 were excluded as irrelevant. The remaining 23 records were assessed for full-text eligibility; 14 were excluded, resulting in 9 studies included in the primary evidence synthesis. The study selection process is summarized in **Figure 1**.

Inclusion criteria were: (1) experimental studies reporting miR-21 activity in human cervical cancer cell lines; (2) studies providing quantitative or semi-quantitative miR-21 expression data from human cervical tissue or patient cohorts; and (3) studies addressing miR-21’s role in EMT, stromal remodeling, fibroblast biology, or related translational applications in cervical cancer. Exclusion criteria were: studies that did not evaluate miR-21 in cervical cancer, did not address EMT, stromal remodeling, fibroblast biology, or related mechanistic pathways, were conducted in non-cervical disease models, or did not provide relevant mechanistic, translational, or clinical outcomes. Case reports, conference abstracts, and editorials were also excluded.

Additional mechanistic and contextual studies identified through reference screening were incorporated narratively, given the conceptual scope of the review. The included studies were heterogeneous in design, comprising *in vitro* cell-line experiments, tissue-based clinical studies, and mixed models, which precluded statistical pooling. Because of this heterogeneity and the hypothesis-generating scope of the review, formal meta-analysis and risk-of-bias appraisal were not performed. Study-level evidence was instead characterized by design type and evidence level—mechanistic/preclinical, translational, or clinical (**Table 1**). Findings from cell-line models are interpreted as mechanistic evidence only and are distinguished throughout from clinical or translational observations.

**Table 1:** Key experimental studies investigating the role of miR-21 in cervical cancer progression, epithelial–mesenchymal transition (EMT), and stromal remodeling.

Author / year	Country	Model / sample	Study type	Main findings	miR-21 target(s)	EMT markers	Clinical / functional outcome	Strength of evidence
Han et al., 2022 (15)	China	HPV16+/18+ cervical cancer cell lines	*In vitro*	HPV infection upregulated miR-21, TGF-βR2, and hTERC	TGF-βR2 / hTERC (co-upregulated)	EMT phenotype (not itemized)	Not assessed	Descriptive (preclinical)
Liu et al., 2020 (16)	China	Cervical cancer cell lines (HeLa, SiHa) + tissue specimens	*In vitro*	Overexpression of miR-21 associated with decreased CRT sensitivity; inhibition of miR-21 reversed EMT markers (↓ vimentin/fibronectin, ↑ E-cadherin).	SMAD7	↓ Vimentin, ↓ Fibronectin, ↑ E-cadherin	miR-21 contributes to therapy resistance via SMAD7/TGF-β signaling; potential predictive biomarker	Translational
Tang et al., 2020 (17)	China	Cervical cancer tissues + lymph node metastases + cell lines (HeLa, SiHa)	Clinical	Higher miR-21 associated with lower E-cadherin and more lymph node metastasis	NS	↑ Vimentin, ↑ N-cadherin, ↓ E-cadherin; ZEB1 inversely correlated with E-cadherin	Correlates miR-21 upregulation and EMT marker shift with metastatic behavior and poor pathology.	Translational
Zhang et al., 2018 (18)	China	Cervical cancer cell lines (HeLa, SiHa) + tissue specimens + serum	*In vitro* + clinical	miR-21 promoted invasion/migration by targeting TIMP3	TIMP3	NS	Links miR-21–TIMP3 to pro-invasive, ECM-remodeling behavior.	Translational
Bumrungthai et al., 2015 (11)	Thailand	Cervical cancer tissues + fibroblast cell line experiments + LCM/ISH	Clinical	miR-21 expressed in stromal fibroblasts; HPV-conditioned media induces miR-21, α-SMA, and IL-6 in fibroblasts with concurrent PDCD4 downregulation	PDCD4 (downregulated); indirect TGF-β/IL-6	↑ α-SMA, ↑ IL-6	Showed stromal source of miR-21, supporting fibroblast activation and fibrosis	Translational
Aguilar et al., 2024 (19)	Mexico	HPV16+ cervical cancer cell lines (CaSki, SiHa)	*In vitro*	miR-21 regulated RECK via binding to its 3′-UTR; low RECK associated with progression and poor chemo response	RECK	NS	miR-21–RECK axis to invasion and chemo response.	Mechanistic (preclinical)
Cai et al., 2018 (20)	China	Cervical cancer cell lines (CaSki, SiHa, HeLa, C4-1, C-33a)	*In vitro*	miR-21-5p upregulated in CC cell lines; overexpression ↑ proliferation/migration/invasion, ↓ apoptosis; VHL is a direct target; VHL knockdown counteracted miR-21-5p inhibition effects	VHL	EMT-like phenotype (↑migration/invasion)	miR-21-5p oncogenic role via VHL suppression.	Mechanistic (preclinical)
Xu et al., 2015 (21)	China	Cervical cancer cell lines (CaSki, HeLa) + tissue specimens	*In vitro*	miR-21 promoted proliferation/migration by inhibiting PTEN	PTEN	NS	Supports miR-21–PTEN axis driving aggressive phenotypes.	Translational
Zhang et al., 2016 (22)	China	89 cervical cancer patients (serum) + cervical cancer cell lines (HeLa and HT-3 cell lines)	*In vitro* + clinical	miR-21 directly targets RASA1 3'-UTR; RASA1 suppression activates RAS/p-AKT/p-ERK signaling and promotes migration; miR-21/RASA1 axis induces EMT	RASA1	↓ E-cadherin, ↓ β-catenin, ↑ N-cadherin, ↑ vimentin	Elevated circulating miR-21 associated with lymph node metastasis (P<0.001) and shorter overall survival	Translational

The table summarizes *in vitro*, *in vivo*, and clinical studies reporting molecular targets of miR-21, associated EMT markers, and functional or clinical outcomes. CRT, chemoradiotherapy; EMT, epithelial–mesenchymal transition; HPV, human papillomavirus; TGF-β, transforming growth factor-beta; hTERC, human telomerase RNA component; ZEB1, zinc finger E-box binding homeobox 1; PDCD4, programmed cell death protein 4; TIMP3, tissue inhibitor of metalloproteinases-3; RECK, reversion-inducing cysteine-rich protein with Kazal motifs; CC, cervical cancer; VHL, von Hippel–Lindau tumor suppressor; siRNA, small interfering RNA; PTEN, phosphatase and tensin homolog; NTF3, neurotrophin-3; α-SMA, alpha-smooth muscle actin; ECM, extracellular matrix; NS, not specified.↓, ↑, means Decreased and Increased.

## 3 Pathophysiology of cervical carcinogenesis

The persistent infection with carcinogenic human papillomaviruses (HPV) has been shown to be the critical cause of ~95% of invasive cervical cancer, including cervical squamous cell carcinoma (SCC) and adenocarcinoma (AC) histotypes (1, 7). Cervical SCC is generally preceded by persistent squamous intraepithelial lesions (SIL) caused by HPV infection; therefore, the detection of viral nucleic acids has been shown to be valuable for the effective prevention of cervical cancer development in oncologic screening programs (1). High-risk HPV oncogenes, particularly *E6* and *E7*, drive cervical carcinogenesis by disrupting p53 and Rb pathways, leading to genomic instability and epigenetic dysregulation (6). The gradual accumulation of genetic and epigenetic alterations in HPV infected cells is also crucial for the ultimate progression to cervical cancer. Epigenetic modifications, including deregulation of microRNA (miRNA) expression have shown to play important roles in cell transformation during distinct stages of cervical intraepithelial neoplasia and cervical carcinoma development (7, 8, 12, 13, 23).

Aberrant miRNA expression is a pivotal characteristic of cervical carcinogenesis, particularly in the context of chronic high-risk HPV infection, where dysregulated miRNAs influence cell cycle regulation, apoptosis, proliferation, invasion, and metastasis by targeting oncogenes and tumor suppressor genes (24–29). Both oncogenic miRNAs — including miR-21, miR-10a, miR-19, and miR-146a — and tumor suppressor miRNAs — including miR-29a, miR-34a, miR-145, and miR-218 — are consistently dysregulated in cervical cancer and associated with disease progression and poor prognosis (25, 30–32). Among these, miR-21 is distinctively positioned: its gene locus at 17q23.2 overlaps a common HPV16 integration site, suggesting that viral integration may directly drive its overexpression, and its simultaneous suppression of multiple tumor suppressors — including PTEN, PDCD4, TIMP3, and VHL — positions it as a convergent oncogenic hub rather than a single-pathway effector (9, 33, 34). Elevated miR-21 expression correlates with progressive histological changes from normal epithelium to advanced cervical cancer, vascular invasion, and lymph node metastasis, and Gebrie et al. (35) identified it as a diagnostic and prognostic biomarker with a sensitivity of 89% and specificity of 79% in a meta-analysis of cervical cancer studies (22, 35).

**Figure 2:**
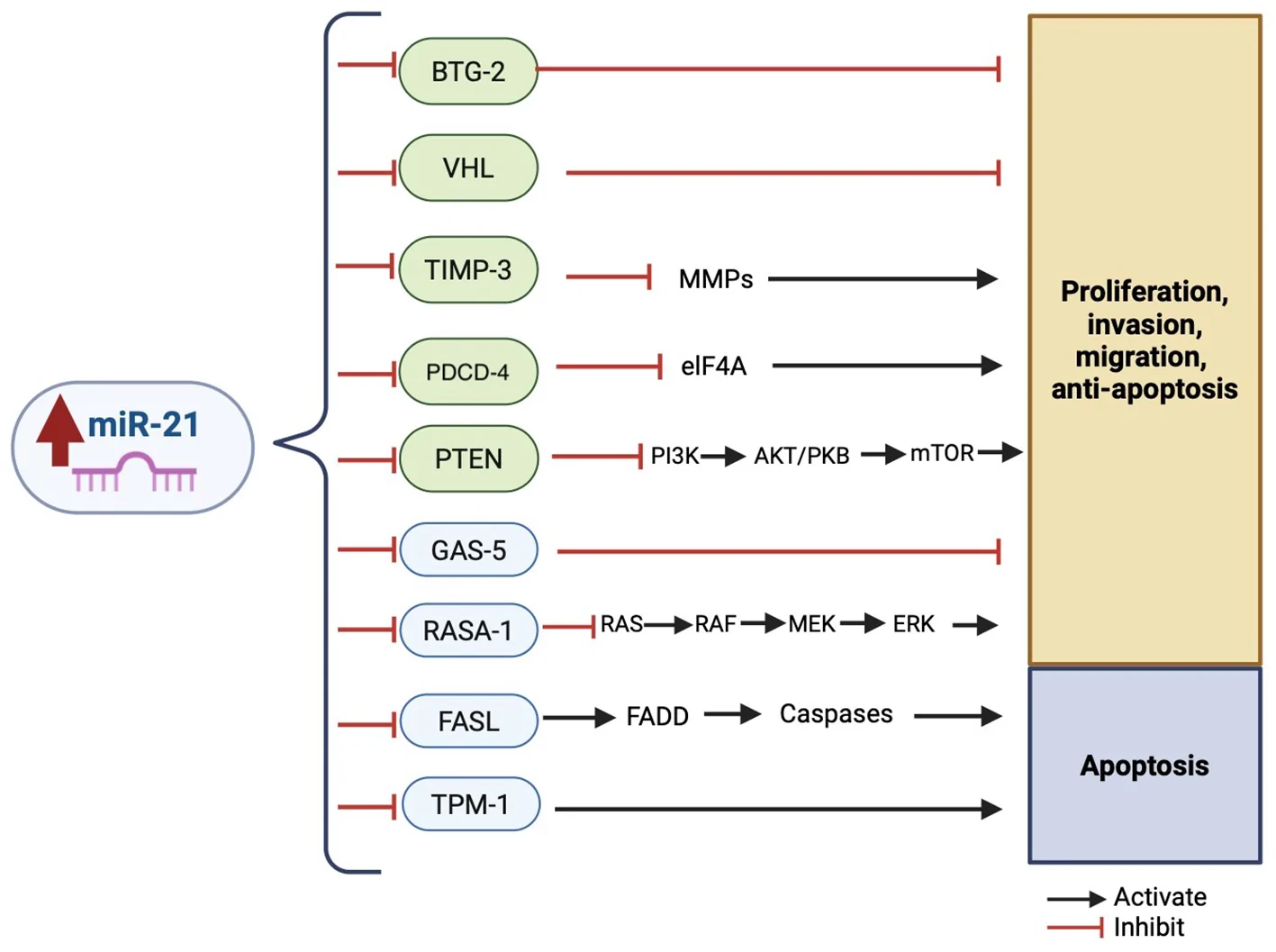
The role of microRNA-21 (miR-21) in the progression of cervical cancer through multiple signaling pathways. These include: PTEN/PI3K/AKT/mTOR, and RASA1/RAS/RAF/MEK/ERK. miR-21 also suppresses tumor suppressor genes such as PDCD4, TPM1, FasL, BTG2, VHL, TIMP3, and GAS5, thus enhancing proliferation, migration, and metastasis. Abbreviations: PTEN, phosphatase and tensin homolog; PI3K, phosphoinositide 3-kinase; AKT/PKB, Akt/protein kinase B; mTOR, mammalian target of rapamycin; RASA1, Ras p21 protein activator 1; RAS, Ras protein; RAF, Raf proto-oncogene; MEK, mitogen-activated protein kinase kinase; ERK, extracellular signal-regulated kinase; PDCD4, programmed cell death 4; eIF4A, eukaryotic initiation factor 4A; TPM1, tropomyosin-1; FasL, Fas ligand; FasR, Fas receptor; FADD, Fas-associated via death domain; BTG2, B-cell translocation gene 2; VHL, von Hippel-Lindau tumor suppressor; TIMP3, tissue inhibitor of metalloproteinases-3; MMPs, matrix metalloproteinases; GAS5, growth arrest-specific 5.

Several reported targets of miR-21 are tumor suppressors, including ligands, receptors, and regulatory proteins involved in key signaling pathways (**Figure 2**). By suppressing TIMP3, a tissue inhibitor of metalloproteinases, miR-21 disturbs the regulation of the extracellular matrix and enhances matrix metalloproteinases (MMP) activity, which leads to more invasion and metastasis. MiR-21 also lowers the levels of some tumor suppressors, including PDCD4, TPM-1, BTG-2, and FasL (35, 36). Inhibition of PDCD4, reduces eLF4A activity, which enhances cell growth, invasion, and metastasis while suppressing apoptosis. Down-regulation of PDCD4 also leads to the inhibition of the inflammatory process through the transcription factor NF-kB and the activation of the anti-inflammatory cytokine interleukin 10 (IL-10), resulting in immune evasion and the progression of cervical cancer (11). Downregulating of PTEN activates Akt/NF-κB signaling, promoting inflammation and tumor progression (10). More broadly, miR-21 has been implicated in promoting Wnt/β-catenin and PI3K/AKT/mTOR signaling in cervical cancer, contributing to tumor proliferation and invasion (27, 37).

miR-21 also promotes cervical cancer progression through the RAS/RAF/MEK/ERK signaling pathway by directly targeting RASA1, a member of the Ras-GTPase-activating protein family that normally suppresses RAS activity (22). Zhang et al. (22) demonstrated, in cervical cancer cell lines and clinical cohort of 89 patients, that miR-21 directly targets the 3’-UTR of RASA1, reducing RASA1 protein levels and activating downstream phospho-AKT and phospho-ERK signaling (22). miR-21-induced RASA1 suppression enhanced cervical cancer cell migration approximately sixfold in transwell assays, an effect reversed by exogenous RASA1 re-expression, and was associated with EMT marker shifts including loss of E-cadherin and gain of N-cadherin and vimentin (22). Clinically, elevated circulating miR-21 in serum was significantly associated with lymph node metastasis in 89 patients with cervical cancer (P<0.001), and higher miR-21 expression correlated with shorter overall survival, supporting the miR-21/RASA1 axis as a clinically relevant mediator of invasion and metastasis (22).

Additionally, miR-21 further promotes cervical cancer by controlling the GAS5 and VHL tumor suppressor pathways. Similarly, suppression of BTG-2, a cell cycle regulator, and FasL, a key inducer of apoptosis in the TNF family, further contribute to tumor progression (35). GAS5, which normally acts through the p53, mTOR, and AKT signaling pathways, has been found to be downregulated in cervical cancer cell lines (35). GAS5 and miR-21 engage in mutual suppression, whereby GAS5 inhibits miR-21 and miR-21 in turn blocks GAS5. Likewise, Cai et al. (20) demonstrated in cervical cell lines that miR-21 directly targets the 3′-UTR of von Hippel–Lindau (VHL) mRNA, confirmed by luciferase reporter assay, suppressing VHL translation and reducing VHL levels, thereby promoting proliferation, invasion, migration, and inhibiting apoptosis (20). Similarly, Chen et al. (38) identified NTF3 as a direct miR-21 target in cervical cancer cell lines, confirmed by luciferase reporter assay, demonstrating that miR-21-mediated suppression of NTF3 promotes proliferation, migration, and invasion while inhibiting apoptosis through PI3K-AKT and MAPK signaling pathways (38). These cell-line observations are consistent with miR-21 participating in GAS5-, VHL and NTF3- the functional consequences for epithelial plasticity and stromal remodeling are discussed in the sections that follow.

## 4 Epithelial–mesenchymal transition as a mechanism of cervical cancer progression

EMT is a process in which epithelial cells lose their apical-basal polarity, undergo cytoskeletal remodeling, and acquire a mesenchymal phenotype characterized by increased motility and invasiveness, apoptosis resistance, and extracellular matrix remodeling. It is triggered by a complex network of signaling factors that activate specific transcription factors, including Snail, Zeb, and Twist, along with miRNAs, epigenetic modifications, and post-translational regulators (16). HPV-driven miR-21 upregulation has been linked to EMT through TGF-β signaling. Elevated TGF-β expression, for example, is strongly associated with progression from cervical intraepithelial neoplasia (CIN) to invasive carcinoma (15). **Table 1** summarizes key *in vitro*, *in vivo*, and clinical studies examining the proposed role of miR-21 in EMT in cervical cancer. Han et al. (15) reported concurrent upregulation of miR-21, TGF-βR2, and hTERC in HPV16+ and HPV18+ cervical cancer cells, suggesting that HPV-driven oncogenic signaling may promote miR-21 upregulation through TGF-β-related pathways and contribute to EMT progression (15).

At the molecular level, *in vitro* studies done by Xu et al. (21) and Liu et al. (16) demonstrated that miR-21 facilitates EMT through two converging mechanisms: suppression of PTEN and PDCD4, which are known to suppress PI3K/Akt, mTOR, and NF-κB cascades; and suppression of Smad7, which is known to inhibit TGF-β/Smad2/3 signaling (16, 21). Both pathways converge on EMT transcription factors including ZEB1, Snail, and Twist (39). Liu et al. (16) and Tang et al. (17) reported downregulation of E-cadherin and upregulation of vimentin, fibronectin, and N-cadherin, facilitating invasion, immune evasion, and resistance to paclitaxel and cisplatin (16, 17). These findings are consistent with the broader literature on EMT in cervical cancer reviewed by Lee and Shen (40), who compiled multiple independent activator categories — including HPV viral proteins, TGF-β and transcription factors such as Snail and Twist — each individually implicated in driving the EMT program in cervical carcinoma (40). This positions miR-21’s target suppression as one contributor within a broader, multifactorial EMT-activating network.

**Figure 3:**
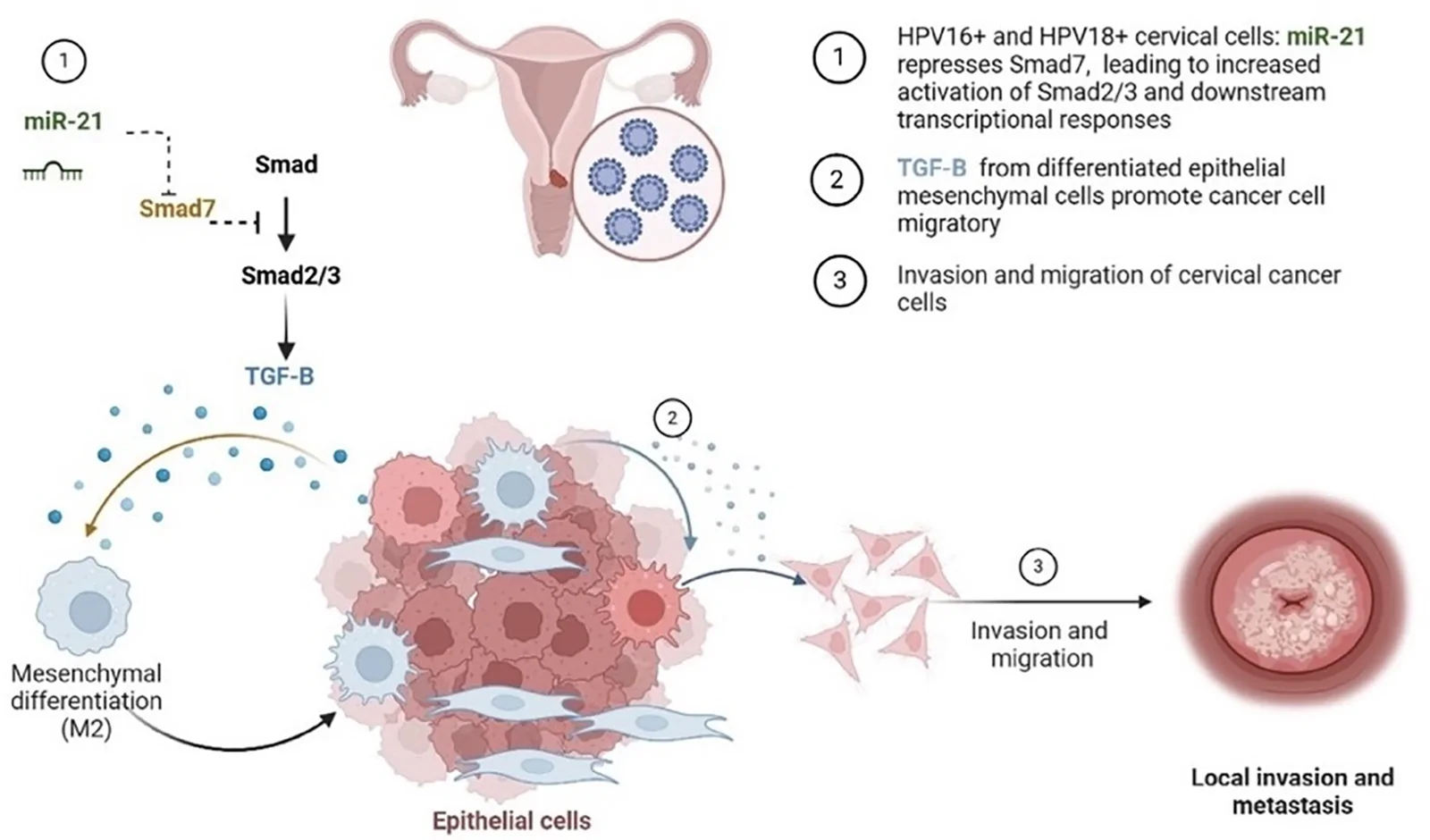
Cellular and molecular changes associated with epithelial–mesenchymal transition (EMT) in cervical cancer. Panel 1 (left): EMT involves loss of apical–basal polarity and acquisition of front-rear polarity, driving the transition from epithelial (E-cadherin–positive) to mesenchymal (N-cadherin–positive, vimentin-rich) phenotypes. Panel 2 (right): In HPV16+ and HPV18+ cervical cancer cells, miR-21 activates Smad2/3 and TGF-β signaling, inducing EMT transcription factors (Snail, Zeb, Twist), fibroblast and mesenchymal stem cell activation, myofibroblast differentiation, and macrophage recruitment, collectively promoting fibrogenesis, invasion, and metastasis.

In cervical cancer tissues, Tang et al. (17) found elevated miR-21 levels in primary cervical carcinoma and lymph node metastases, with parallel overexpression of ZEB1 and Snail in tumors with deeper muscle infiltration and greater nodal involvement. ZEB1 protein levels were inversely correlated with E-cadherin, consistent with its established role as a transcriptional repressor (17). A functionally important consequence of miR-21-mediated EMT is the acquisition of chemoradiotherapy resistance. Liu et al. (16) demonstrated that miR-21 overexpression in advanced cervical cancer cells reduced sensitivity to chemoradiotherapy, and that miR-21 inhibition reversed this effect — restoring E-cadherin and reducing vimentin and fibronectin — through a Smad7-dependent mechanism (16, 33). When Smad7 was also inhibited, the resensitization effect was diminished, implicating the miR-21/Smad7/TGF-β axis as a regulator of both EMT and treatment resistance (16) (**Figure 3**). Converging evidence from other solid tumors confirms Smad7 as a direct miR-21 target in TGF-β/Smad signaling dysregulation (41).

**Figure 4:**
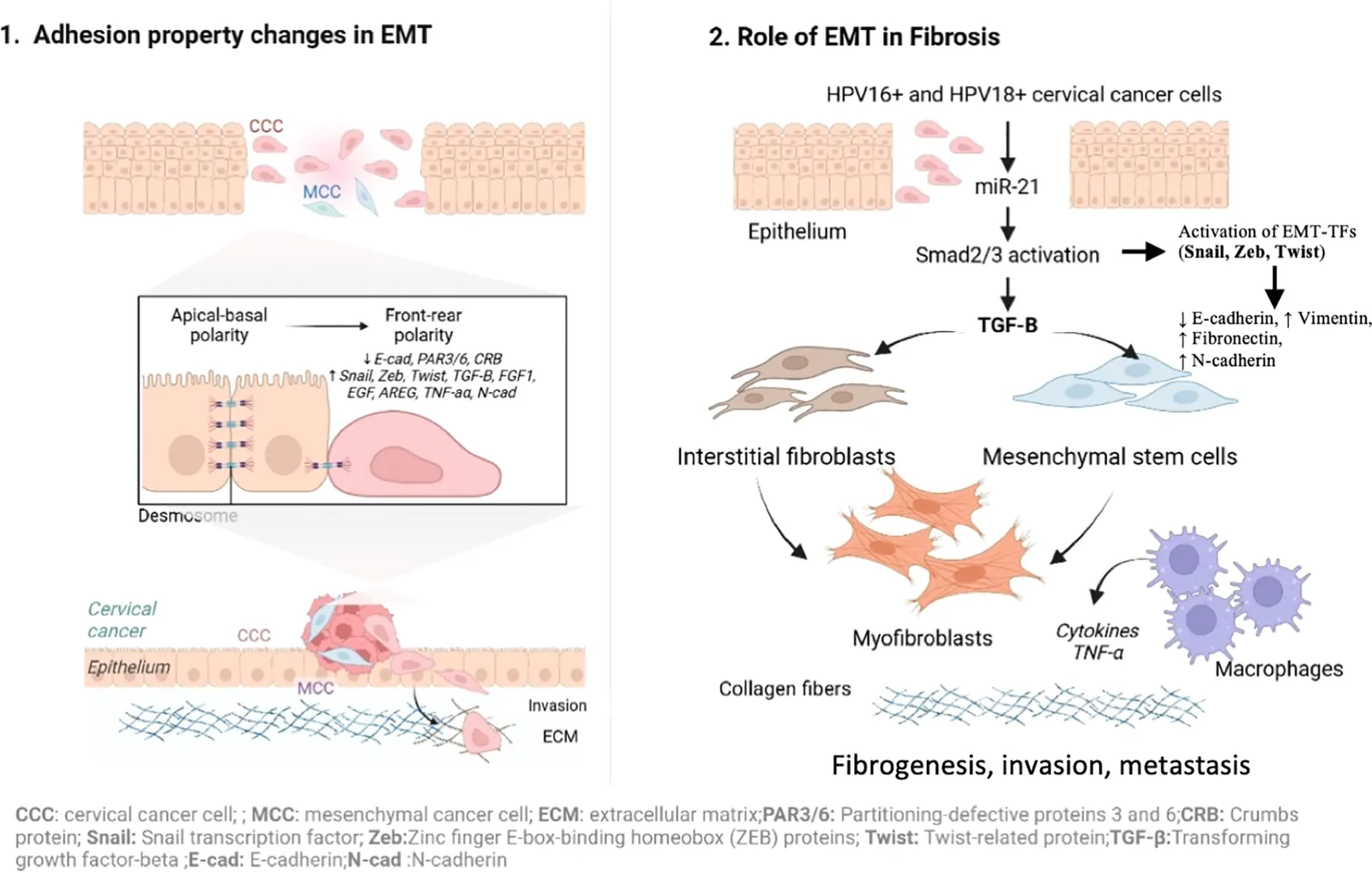
miR-21–mediated oncogenic pathway in cervical cancer progression. In HPV16+ and HPV18+ cervical cancer cells, miR-21 suppresses Smad7, enhancing TGF-β/Smad2/3 signaling and EMT. This drives epithelial cells to undergo mesenchymal differentiation, resulting in loss of adhesion and increased migratory potential. The secreted TGF-β further promotes a tumor-supportive microenvironment, amplifying invasion and migration. Ultimately, this miR-21–mediated pathway contributes to local invasion, lymph node metastasis, and therapy resistance, underscoring miR-21 as a potential biomarker and therapeutic target.

Building on the previous findings, we propose a model in which miR-21 activates Smad2/3 signaling, driving TGF-β-mediated fibroblast activation and EMT transcription factor induction — a synthesis depicted in **Figure 4**. Although the evidence suggests a role for miR-21 in EMT-related pathways, the strength of evidence varies considerably across studies. Most mechanistic data originate from cervical cancer cell lines, where miR-21 overexpression or inhibition directly alters EMT markers and invasive behavior. These experimental models permit causal inference regarding molecular pathways but may not fully recapitulate the complexity of the human tumor microenvironment. In contrast, clinical studies primarily demonstrate associations between elevated miR-21 expression, altered EMT marker profiles, lymph node metastasis, and adverse pathological features (17, 22). While these observations are consistent with the proposed mechanistic role of miR-21, they do not independently establish causality. Therefore, current evidence supports miR-21 as a plausible regulator of EMT in cervical cancer, but prospective validation in patient-derived models and clinical cohorts remains necessary.

## 5 Mechanistic role of miR-21 in stromal remodeling

EMT plays a key role in various types of fibrosis, including the lung, liver and kidneys. EMT-related transcription factors (EMT-TFs) are essential for the process to develop. For example, renal epithelial cells that undergo a partial EMT and lose their normal function, release paracrine signals to the renal interstitium, reshaping the local environment (41). TGFβ signaling drives the differentiation of fibroblasts into activated myofibroblasts, while concurrent cytokine and chemokine secretion promotes macrophage infiltration into the stroma, collectively amplifying the fibroinflammatory microenvironment (41).

miR-21 has been proposed as a central regulator of fibrotic diseases in other organ systems, promoting fibroblast proliferation and extracellular matrix deposition (42). Huang et al. (42) reviewed the role of miR-21 across fibrotic diseases and reported that miR21 is persistently elevated in fibrotic tissues, where it facilitates fibroblast proliferation and excessive extracellular matrix deposition, two prominent characteristics of fibrosis (42). While Huang et al.’s review was not specific to cervical cancer, its characterization of miR-21 as a profibrotic regulator provides biological plausibility for a similar role in the cervical tumor microenvironment. Likewise, the mechanistic link between miR-21, PDCD4 suppression, and myofibroblast differentiation was demonstrated by Yao et al. (36) in a non-cervical tumor-stroma model, where miR-21 directly targeted the 3’-UTR of PDCD4 in fibroblasts stimulated by TGF-β1 or cancer cell-conditioned medium, promoting fibroblast-to-myofibroblast differentiation (36). While this study was also not conducted in cervical cancer, it provides mechanistic context for the proposed role of miR-21 in fibroblast-to-myofibroblast differentiation within the tumor stroma.

Consistent with this, Bumrungthai et al. (11) demonstrated miR-21 upregulation in cervical tumor-associated fibroblasts accompanied by increased α-SMA expression, suggesting that fibroblast activation — a hallmark of stromal remodeling — may represent a miR-21-mediated process in cervical cancer, though direct histopathologic evidence of fibrosis in this context remains lacking (11). Bumrungthai et al. (11) found that HPV-driven miR-21 upregulation in cervical tumor-associated stromal fibroblasts was accompanied by concurrent PDCD4 downregulation and increased α-SMA and IL-6 expression, consistent with fibroblast activation (11). Collectively, these predominantly cell-line findings are consistent with miR-21 contributing to fibroblast-to-myofibroblast differentiation in the tumor stroma, which may facilitate tumor progression. Activated fibroblasts may also act on tumor cells through expression of growth factors such as TGF-β1, contributing to tumor cell survival and proliferation. TGF-β1 activates the Erk-MAP kinase pathway, leading to AP-1 expression, a known transcriptional activator of the miR-21 promoter in stromal fibroblasts and cancer cells (11). This regulatory loop — in which TGF-β1 and IL-6 induce miR-21, which in turn amplifies fibroblast activation — means that the existing experimental evidence does not determine whether miR-21 is a primary driver of stromal remodeling or whether its upregulation in fibroblasts is itself a consequence of the broader inflammatory milieu. TGF-β, IL-6, and AP-1 are each independently capable of driving myofibroblast differentiation and ECM remodeling, and the current literature does not establish that miR-21 is a necessary driver of stromal remodeling in cervical cancer, as opposed to one component of a broader fibroinflammatory response.

TIMP3, introduced above as a miR-21 target that enhances MMP activity, contributes to stromal remodeling through excessive ECM degradation. Zhang et al. (18) demonstrated in cervical cancer cell lines and tissue specimens that miR-21 directly targets and suppresses TIMP3, thereby enhancing MMP activity and promoting ECM degradation, cell migration, and tumor invasion (18). This suppression of TIMP3 not only increases metastatic potential but may also contribute to stromal remodeling through excessive ECM degradation (18). Consistent with this, Tang et al. (17) reported that miR-21 and ZEB1 expression were significantly elevated in cervical cancer and lymph node metastasis tissues compared with normal cervical tissue, with miR-21 positively correlated with ZEB1 (rs=0.841, P<0.05) and ZEB1 inversely correlated with E-cadherin protein expression (rs=-0.862, P<0.05) (17). In HeLa and SiHa cell lines, miR-21 overexpression upregulated vimentin and N-cadherin and downregulated E-cadherin, consistent with an EMT phenotype, and enhanced cellular invasiveness for which direct histopathologic evidence in cervical cancer is currently lacking (17). That mesenchymal marker expression and reduced epithelial markers, suggest a possible role in stromal remodeling for which direct histopathologic evidence in cervical cancer is currently lacking.

Additionally, Aguilar et al. (19) studied HPV16+ cervical cancer cell lines and found that miR-21 primarily regulates the expression of the RECK gene through MRE21-1, MRE21-2, and MRE21-3, which are situated within the RECK 3′-UTR (19). RECK is a glycoprotein that is attached to the cell membrane and binds to and negatively controls several MMPs. These MMPs break down extracellular matrix proteins that are important for tumor invasion and metastasis. Lower RECK mRNA levels are associated with cervical lesion progression and poor response to chemotherapy in cervical cancer patients (19). These *in-vitro* results identify a regulatory network involving miR-21 and the RECK in HPV-transformed cervical cells; clinical validation of this axis is needed.

The evidence supporting a role for miR-21 in stromal remodeling is less directly supported by cervical-specific data than the evidence for its involvement in EMT. Much of the proposed mechanism is extrapolated from fibrosis studies in non-cervical tissues and experimental fibroblast models. Although cervical cancer studies demonstrate increased miR-21 expression in tumor-associated fibroblasts and suggest links with fibroblast activation, it is important to note that increased α-SMA and IL-6 expression in fibroblasts represent fibroblast activation — a cellular phenotype consistent with, but not equivalent to, histologically confirmed stromal fibrosis. The proposed fibrosis-like phenotype in cervical cancer is therefore currently inferred from fibroblast activation markers in experimental models rather than directly demonstrated in cervical cancer tissue specimens. Direct histopathologic evidence correlating miR-21 expression with stromal fibrosis, extracellular matrix remodeling, or clinical outcomes in cervical cancer patients remains limited. Consequently, the proposed fibrosis-like role of miR-21 in the cervical tumor microenvironment should currently be viewed as a biologically plausible but incompletely validated hypothesis — inferred from fibroblast activation markers in experimental models rather than directly demonstrated through histopathologic assessment of stromal fibrosis or extracellular matrix remodeling in cervical cancer tissue.

## 6 Therapeutic targeting of miR-21

Several therapeutic agents targeting miR-21 have been studied in preclinical and early-phase research studies (**Table 2**). Because few of these agents have been evaluated specifically in cervical cancer models, this section draws primarily on the broader mechanistic and therapeutic literature rather than the nine studies identified through the search described above. PLGA-Curcumin Nanoparticles (Nano-CUR) represent the most advanced anti-miR-21 strategy tested specifically in a cervical cancer *in vivo* model (43). Zaman et al. demonstrated that Nano-CUR significantly reduced tumor burden in a preclinical orthotopic mouse model of cervical cancer by decreasing oncogenic miR-21, suppressing nuclear β-catenin, and abrogating E6/E7 HPV oncoprotein expression (44). *In vitro*, Nano-CUR outperformed free curcumin in inhibiting cell growth, inducing apoptosis, and arresting the cell cycle in cervical cancer cell lines (44). This is the only anti-miR-21 approach with both cervical cancer-specific *in vivo* efficacy data and a defined mechanism linking miR-21 suppression to HPV oncoprotein modulation. However, the effect on miR-21 is indirect (via STAT3 pathway disruption), and the specificity of curcumin’s miR-21 effects has been questioned—one study in HPV16-positive CaSki cells found no significant change in miR-21 after curcumin treatment, while another in SiHa cells showed dose-dependent miR-21 reduction, suggesting cell-line and context dependence (43).

**Table 2:** Preclinical and experimental therapeutic strategies targeting miR-21 in cervical cancer and other solid tumors.

Therapeutic strategy	Mechanism of action	Preclinical/experimental effects	Stage
Synthetic oligonucleotides (e.g., antagomirs, antisense oligos) (45, 60–62)	Bind/block miR-21 or disrupt gene locus → restore tumor suppressor activity(e.g., PTEN/PDCD4)	↓ Proliferation/colony formation, ↑ paclitaxel sensitivity, restored tumor suppressor activity	*In vitro*
TALEN-mediated gene disruption (62)	Disrupt miR-21 gene locus → permanent loss of miR-21 expression	↓ Proliferation, ↓ anchorage-independent growth, ↑ cisplatin sensitivity	*In vitro* + *In vivo* (xenograft)
Small-molecule inhibitors (e.g., amide/aryl amide derivatives, AC1MMYR2) (46–49)	Inhibit Dicer-mediated miR-21 biogenesis or directly reduce miR-21 expression	↓ miR-21, reversed EMT, ↓ invasion/tumor growth	*In vitro* + *In vivo* (mouse models)
Natural compounds (e.g., curcumin, curcumol) (43, 44)	Downregulate miR-21 expression	↓ Proliferation, migration, tumor burden	*In vitro* + *In vivo* (animal models)

↓, ↑, → means Decreased and Increased.

Antisense miR-21 inhibitors (standard ASOs) are the most widely validated modality across cervical cancer cell lines. Du et al. (45) showed that miR-21 inhibition suppressed proliferation, induced apoptosis, upregulated PTEN and PDCD4, and increased paclitaxel sensitivity (45). Small-molecule inhibitors, including amide and aryl amide derivatives and AC1MMYR2, inhibit Dicer-mediated processing of pre-miR-21, resulting in reduced levels of mature miR-21, reversal of EMT markers, and decreased tumor growth in preclinical studies (46–49). Additionally, DNAzyme-based molecules designed to selectively inhibit miR-21 in solid tumors can be combined with aptamers to improve delivery but are experimental and untested in cervical models (50). Despite this breadth of approaches, standard ASOs lack cervical cancer-specific *in vivo* data, and the most pharmacologically advanced direct inhibitors — mesyl phosphoramidate oligonucleotides and AC1MMYR2 — have demonstrated *in vivo* efficacy in other solid tumors but have not been tested in cervical cancer models, representing an unaddressed translational gap (46–49).

The absence of clinical translation reflects barriers that are shared across all anti-miR-21 modality classes. miR-21’s broad expression across normal tissues means that systemic inhibition risks modulating gene networks beyond the tumor, with unpredictable off-target consequences that standard cell-line and xenograft models cannot anticipate (51–53). Delivery is a fundamental obstacle of oligonucleotide-based approaches, which face rapid systemic degradation, preferential accumulation in liver and kidney rather than solid tumors, and limited endosomal escape after cellular uptake (54–56). The relative clinical advancement of anti-miR-21 therapy in Alport nephropathy — where miR-21 is activated selectively after renal injury and oligonucleotides naturally accumulate in the kidney — illustrates how disease-specific biology and delivery alignment can enable translation, advantages that do not currently exist for cervical cancer (57). Until tumor-selective delivery strategies and validated pharmacodynamic biomarkers are established, the therapeutic potential of miR-21 inhibition in cervical cancer will remain preclinical.

## 7 Limitations and future directions

The majority of the evidence derives from *in vitro* and clinical studies conducted predominantly in Asian populations, limiting the generalizability of the findings. This highlights the need for validation across different populations, as regional genetic and environmental differences may influence miRNA expression and cancer biology. For example, HPV-16 accounts for approximately 57-62% of invasive cervical cancers worldwide, with the highest regional prevalence in western-central Asia (73%) and lowest in Africa (53%) (9, 34). Because the mechanistic studies reviewed here relied predominantly on HPV16+ and HPV18+ cell lines, the identified miR-21 targets and downstream pathways may not fully represent cervical cancers driven by other high-risk subtypes — such as HPV45, HPV31, and HPV33 — which contribute more substantially to disease burden in sub-Saharan Africa and Latin America (34). In the United States, HPV-16 prevalence is 1.5% overall among females aged 14–59 years, with higher rates in younger women: 9.6% in those ≤20 years, 6.5% in ages 21-29, and 1.8% in those ≥30 years (58). Additionally, the incidence rates of cervical squamous cell carcinoma are highest in African American and Hispanic women, with Black women having the highest overall mortality rate, irrespective of subtype or stage (59).

A related methodological limitation is the absence of a formal risk-of-bias or quality appraisal of the included studies. Because the nine identified studies are heterogeneous in design, spanning preclinical, translational, and clinical models, no single appraisal instrument applies uniformly across all study types, and applying separate tools to each design subgroup was judged unlikely to yield results that were meaningfully comparable given the narrative scope of the review. Instead, each study was characterized by design type and evidence level (**Table 1**), allowing readers to contextualize individual findings without implying comparability across studies that were not intended to be pooled. This reflects a deliberate methodological trade-off: PRISMA-style identification was retained to ensure transparency of the core evidence base, while formal risk-of-bias appraisal was not undertaken given the review’s narrative, hypothesis-generating aims. Consequently, the relative methodological strength of the nine included studies could not be systematically weighted, and conclusions drawn from this evidence base should be interpreted accordingly. Finally, the interpretation of findings across studies can also be limited by publication bias.

## 8 Conclusion

The existing evidence from *in vitro* and clinical studies suggests that miR-21 may function as an oncogenic mediator in cervical cancer at the intersection of EMT and stromal remodeling. Driven by HPV E6/E7 oncoproteins, miR-21 suppresses key tumor suppressors and activates downstream cascades associated with EMT, fibroblast activation, and extracellular matrix remodeling, with preliminary evidence also linking miR-21 to reduced chemoradiotherapy sensitivity. Stromal remodeling may represent not merely a downstream consequence of EMT, but a potentially miR-21-mediated process that amplifies tumor aggressiveness through myofibroblast differentiation and paracrine signaling. Taken together, these findings support a hypothesis-generating framework rather than an established causal pathway.

In addition, most of the available evidence derives from *in vitro* studies conducted in Asian populations, which limits the generalizability of the findings. Unlike previous reviews that have focused primarily on miR-21 from a proliferative or metastatic perspective, this synthesis highlights its proposed role in fibroblast activation and stromal remodeling as a biologically plausible but not yet directly validated oncogenic mechanism. Future research should integrate molecular, histologic, and clinical parameters to determine whether targeting miR-21, alone or in combination with anti-fibrotic therapies, can improve diagnosis, prognosis, and treatment outcomes in cervical cancer.
